# Phenol adsorption on high microporous activated carbons prepared from oily sludge: equilibrium, kinetic and thermodynamic studies

**DOI:** 10.1038/s41598-019-55794-4

**Published:** 2019-12-18

**Authors:** N. Mojoudi, N. Mirghaffari, M. Soleimani, H. Shariatmadari, C. Belver, J. Bedia

**Affiliations:** 1 Department of Natural Resources, Isfahan University of Technology, Isfahan, 84156-83111 Iran; 2 Department of Soil Science, College of Agriculture, Isfahan University of Technology, Isfahan, 84156-83111 Iran; 3 Departamento de Ingeniería Química, Facultad de Ciencias, Universidad Autónoma de Madrid, Cantoblanco, 28049 Madrid, Spain

**Keywords:** Environmental chemistry, Engineering

## Abstract

The purpose of this study was the preparation, characterization and application of high-performance activated carbons (ACs) derived from oily sludge through chemical activation by KOH. The produced ACs were characterized using iodine number, N_2_ adsorption-desorption, Fourier-transform infrared spectroscopy (FTIR) and scanning electron microscopy (SEM). The activated carbon prepared under optimum conditions showed a predominantly microporous structure with a BET surface area of 2263 m^2^ g^−1^, a total pore volume of 1.37 cm^3^ g^−1^ and a micro pore volume of 1.004 cm^3^ g^−1^. The kinetics and equilibrium adsorption data of phenol fitted well to the pseudo second order model (R^2^ = 0.99) and Freundlich isotherm (R^2^ = 0.99), respectively. The maximum adsorption capacity based on the Langmuir model (434 mg g^−1^) with a relatively fast adsorption rate (equilibrium time of 30 min) was achieved under an optimum pH value of 6.0. Thermodynamic parameters were negative and showed that adsorption of phenol onto the activated carbon was feasible, spontaneous and exothermic. Desorption of phenol from the adsorbent using 0.1 M NaOH was about 87.8% in the first adsorption/desorption cycle and did not decrease significantly after three cycles. Overall, the synthesized activated carbon from oily sludge could be a promising adsorbent for the removal of phenol from polluted water.

## Introduction

The quality of water is deteriorating exponentially due to the growth of world population, industrialization, rapid urbanization and extensive human activities^1^. Discharging of untreated or inadequately treated wastewater of various industries into the natural environment leads to water pollution. Phenol and phenolic compounds are highly carcinogenic and have been classified as priority pollutants by the US Environmental Protection Agency (EPA) because of their high toxicity even at low concentrations^2^.

The most common methods for removal of phenol from aqueous solutions include extraction, distillation, ion exchange, sedimentation, chemical oxidation, reverse osmosis processes as well as adsorption. Among these physicochemical processes, adsorption has been widely used due to its low cost of implementation, wide range of applications, high efficiency and easy operational design^2–4^.

Activated carbon (AC) is the most common adsorbent in water and wastewater treatment^5,6^. AC is a carbonaceous material with a well-developed porous structure and large specific surface area that can be produced from different precursors with high carbon content and preferably low amount of inorganic content^6^. Usually, commercial ACs are expensive because of the relatively high-cost of raw materials^5^. One alternative to reduce the ACs cost is the usage of cheap and widely available materials such as different solid wastes^7,8^. Available industrial wastes and low-cost materials are among the most interesting precursors for preparation of ACs^9^. Oily sludge can be used as a precursor for AC preparation due to its considerable carbon content. The use of oily sludge for the production of carbonaceous adsorbents, despite scarcely investigated, could be an interesting technique for the management of this residue from an economic and environmental points of view.

Significant volume of oily sludge as a waste is generated unavoidably in oil refinery wastewater treatment plants, storage tanks of crude oil, petroleum production and crude oil transportation facilities. Disposal of oily sludge can lead to a multitude of serious environmental problems^10^. Handling, treatment and safe disposal of enormous quantity of oily sludge are considered as a major challenge facing hazardous waste management in oil industries. From an environmental point of view, the efficient and optimum use of oily sludge is the best strategy for its treatment^11^.

This work presents the production of ACs from oily sludge using two steps including thermal and thermochemical treatments for improvement the process. Optimization of the synthesis conditions was also performed to reduce the consumed chemicals and reagents. To our knowledge, only two previous studies reported the synthesis of AC from oily sludge, and none of them optimized the preparation conditions, resulting in ACs with relatively low specific surface area^11^ or needed of a de-oiling step before activation^12^. In this study, a more convenient technique was applied for the purpose of preparing ACs with high surface areas. The produced ACs were repeatedly washed with hot and cold water followed by acid washing. Acid washing and creation of thermal stress can be useful for enhancing porosity as well as specific surface area. Furthermore, the potential of the AC with the highest specific surface area was investigated for removal of phenol from aqueous solutions. According to our knowledge and literature review, phenol removal using an AC derived from oily sludge has been not reported. Besides, equilibrium, kinetic, thermodynamic and regeneration studies of phenol adsorption by the synthesized AC were also investigated. Thus, the novelty of this study is the synthesis of ACs with very well-developed porous texture through chemical activation by KOH of oily sludge residue devoted to phenol adsorption from water.

## Results and Discussion

### Characteristics of oily sludge

The amount of C, H, N, S and O were 82.4, 12.5, 0.2, 1.6, and 3.3 percent, respectively. Oily sludge presented relatively low ash content of 6.2%. The precursors for the synthesis of activated carbons should be preferably available, cheap, high in carbon and low in ash content^13^. The high amount of carbon (82.4%) and the relatively low ash content (6.2%) suggest that oily sludge can be considered as a valuable material for preparation of AC, if an appropriate method is used.

### Characteristics of activated carbons

It can be clearly observed from Table 1 that the iodine number of ACs increases with increasing the activation temperature from 600 to 800 °C and the impregnation ratio from 1:1 to 2:1. According to the obtained results, the highest porosity was obtained at an activation temperature of 800 °C and an impregnation ratio of 2:1. Activation temperature plays an important role in the formation of micro- or mesopores in the ACs^14^. Generally, an increase of activation temperature from 600 °C to 800 °C, resulted the development of higher porous structure. This could be due to the reaction of KOH and carbon surface, vaporization of volatile material and development of new pores in the structure of raw materials^15^. However, a further increase in the activation temperature up to 900 °C led to a contraction of the porous structure. Small amounts of metallic potassium are produced at temperatures higher than 700 °C that intercalate between the carbon layers. Excessive temperature may cause the destruction of pore walls and transform micropores into meso- or macropores^16^, resulting in a porosity loss in agreement with the results of this work. Furthermore, it is also reported that very high temperatures increase the rate of gasification reaction resulting in destruction of micropores and reducing the surface area of AC^17^. The increase of the impregnation ratio from 1:1 to 2:1 resulted in a porosity development due to the presence of more KOH that could react with the carbon surface causing more release of gases and formation of more volume of pores on surface of adsorbent. However at higher impregnation ratios, the micropores might coalescence as a result of the deeper carbon decomposition^18^. At the highest impregnation ratio of 3:1, pore volume decreased as a consequence of destructive effect of chemicals on the micropore structure as reported by Yahya *et al*.^19^.

**Table 1:** Iodine number, porosity characteristics and yield of the produced activated carbons from oily sludge by KOH activation.

Sample	S_BET_ (m^2^ g^−1^)	S_mic_ (m^2^ g^−1^)	S_ext_ (m^2^ g^−1^)	V_total_ (m^3^ g^−1^)	V_mic_(m^3^ g^−1^)	V_mic_ (%)	Iodine number	Yield (%)
ORS char	14	—	14	0.04	—	—	8.4	13.1
CK1T600	228	167	61	0.205	0.078	36.58	248.34	34.77
CK1T700	427	332	94	0.337	0.156	46.29	403.82	30.62
CK1T800	1334	1022	312	1.04	0.48	46.15	1023.44	28.21
CK1T900	1236	931	305	0.98	0.441	45	916.76	24.46
CK2T600	448	341	107	0.365	0.161	44.1	461.68	30.87
CK2T700	1118	857	261	0.872	0.406	46.55	801.85	26.37
CK2T800	2263	2045	218	1.37	1.004	73.28	2003.87	25.17
CK2T900	1680	1306	391	1.33	0.621	46.69	1242.88	23.41
CK3T600	312	191	121	0.28	0.091	32.5	376.23	29.45
CK3T700	1080	727	354	0.81	0.353	43.58	762.53	25.48
CK3T800	1751	1587	164	1.05	0.777	74	1488.02	24.12
CK3T900	1425	1087	356	1.17	0.51	43.58	1081.15	22.81

C: Activated Carbon K1: ratio of KOH to Carbon = 1, K2: ratio of KOH to Carbon = 2, K3: ratio of KOH to Carbon = 3, T600 = Activation temperature of 600 °C, T700 = Activation temperature of 700 °C, T800 = Activation temperature of 800 °C, T900 = Activation temperature of 900 °C.

**Figure 1:**
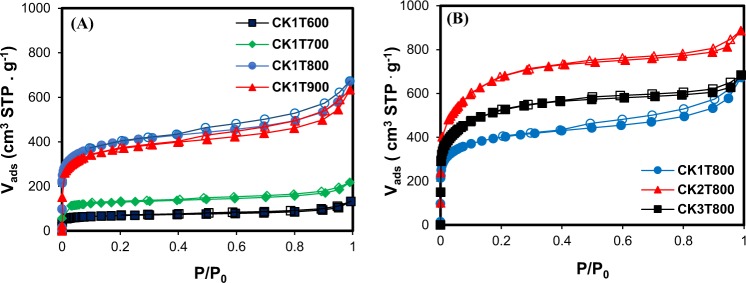
N_2_ adsorption/desorption isotherms at −196 °C of the prepared activated carbons by KOH from oily sludge (CK) at (**A**) different activation temperatures (T, 600, 700, 800 and 900 °C) and at impregnation ratio of 1:1 (KOH to carbon), and (**B**) different impregnation ratios (1:1, 2:1 and 3:1) and an activation temperature of 800 °C.

The N_2_ adsorption/desorption isotherms of ACs prepared at different activation temperatures and an impregnation ratio of 1:1 are shown in Fig. 1(A). The adsorption isotherms for all those ACs belong to the combination of type І and type ІV isotherms according to the IUPAC classification^20^, which indicates the existence of both micro- and mesopore structures. According to the plots, with increasing the temperature from 600 to 800 °C, the amount of N_2_ adsorbed at low relative pressures increases demonstrating the higher microporosity of the produced activated carbon^20^. Type І isotherm is typically attributed to the microporous samples with a relatively small external surface, while some mesopores exist in the AC due to slight increase of N_2_ adsorption at higher relative pressure after filling the micropores^21^. After progressive increasing of P/P_0_ in the isotherm of type I, a roughly horizontal line extended up. This isotherm describes the monolayer adsorption, which is consistent with the Langmuir equation^21^. Figure 1(B) shows N_2_ adsorption/desorption isotherms of the obtained ACs with different impregnation ratios and at activation temperature of 800 °C. All the ACs showed again a combination of type I and type ІV isotherms of the IUPAC classification demonstrating the co-existence of micro and mesopores in the structure of ACs. The hysteresis loops in all desorption isotherms are characteristics of the existence of slit-shaped micropores and/or mesopores in the structure of these samples^22^. The maximum percentage of micropore volume (73%) was obtained at an impregnation ratio of 2:1 (mass ratio of activating agent to carbonized oily sludge) and an activation temperature of 800 °C. This sample (CK2T800), which shows the maximum BET value of 2263 m^2^ g^−1^ and iodine number of 2003 mg g^−1^, was selected for further characterization.

**Figure 2:**
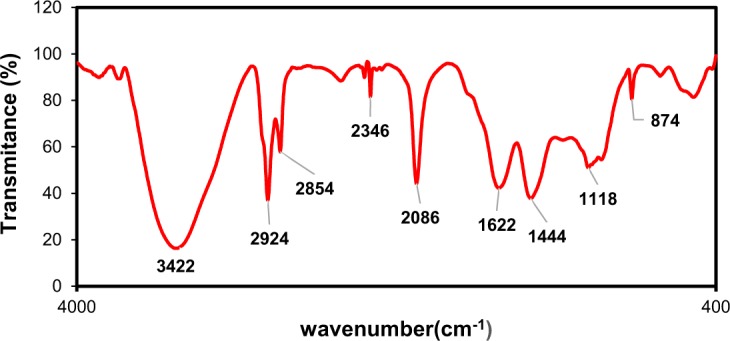
FT-IR spectra of the prepared activated carbon at temperature of 800 °C and impregnation ratio of 2:1 (KOH to carbon).

FTIR analysis was used for identifying surface functional groups of the activated carbon. Figure 2 represents the FT-IR spectrum of CK2T800 sample. The broad peak around 3422 cm^−1^ is associated to the bands of O–H group due to the vibration of water molecules. The noticeable peak at 2924 cm^−1^ is attributed to presence of aliphatic C–H stretch of CH, CH_2_ and CH_3_ groups^23^. The peak at 2854 cm^−1^ is assigned to the CH_2_ symmetric stretching. The peak observed at 2345 cm^−1^ arises due to the presence of C = C groups. The peak at 2085 cm^−1^ is related to the C = N stretching vibrations^24^. The peak which is presented at 1622 cm^−1^ can also be corresponded to C = O stretching of carboxylic acids^25^. The band 1444 cm^−1^ assigns to C-H asymmetric and symmetric bending vibrations^26^. The weak band in the range between 900 to 1300 cm^−1^ may be due to the presence of C–O group in the sample. The band found at 874 cm^−1^ is related to the stretching vibrations of C−H out-of-plane band^27^.

**Figure 3:**
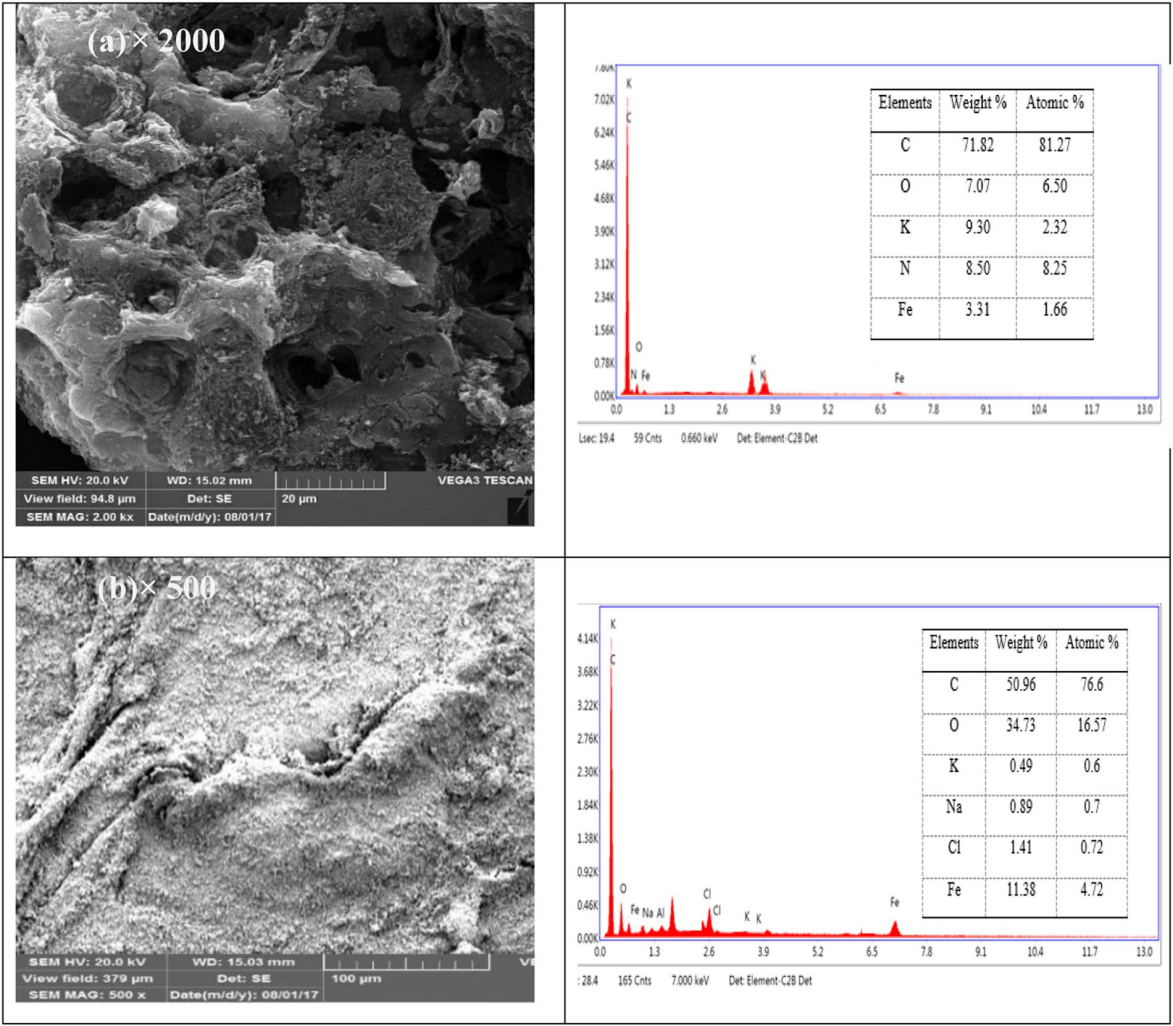
SEM micrographs and EDX analysis of (**a**) the prepared activated carbon at temperature of 800 °C and impregnation ratio of 2:1 (KOH to carbon) and (**b**) char produced from oily sludge.

The morphological structures of the oily residue sludge char and CK2T800 activated carbon were analyzed by SEM. SEM images revealed that the external surface of activated carbon was very irregular and full of cavities with different shapes and sizes comparing to that of the non-activated char. This can be a consequence of the gasification and release of volatile matter produced during the activation process (Fig. 3). EDX analysis indicated the presence of C and O in the prepared AC. The atomic percentage of C and O calculated from the quantification of the peaks, gave the value of about 81 and 6, respectively. This relatively high carbon content and the absence of alkali and alkaline earth metal indicated that the carbonization process and the washing steps were successful.

### Phenol adsorption

**Figure 4:**
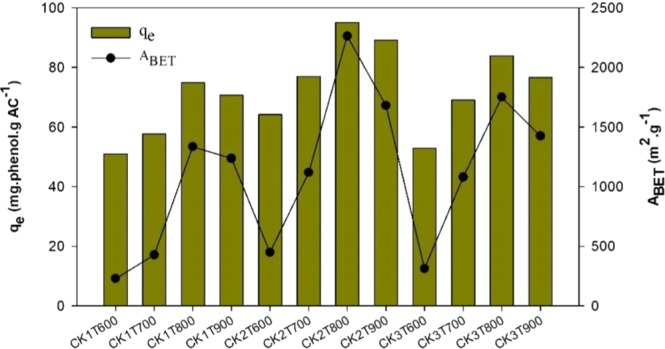
Equilibrium phenol adsorption capacities (q_e_) and BET surface area (A_BET_) of different prepared activated carbons by KOH (CK) at different activation temperatures (T, 600, 700, 800 and 900 °C) and different impregnation ratios (KOH to carbon) of 1:1, 2:1 and 3:1 **(**T = 25 °C; contact time = 4 h; initial concentration of phenol = 100 mg L^−1^; sorbent dose = 1 g L^−1^).

Initial adsorption tests were performed with all the synthesized ACs in order to select the one with the most suitable properties for the phenol removal from aqueous phase. Figure 4 shows the amounts of phenol adsorbed (mg g^−1^) by the different ACs and their BET surface areas. It can be observed a relation between the BET values and the adsorption capacities of the activated carbons^28^. As shown in Fig. 4, the highest phenol adsorption was obtained with CK2T800 activated carbon, which is the AC with the highest surface area and more developed porous structure. Therefore, this carbon was selected for the subsequent adsorption experiments. Furthermore, it should be mentioned that the adsorption capacity of phenol on the non-activated char was only 14.28 mg g^−1^, which was significantly lower than of activated carbons in agreement with its much lower porosity development. This confirms that the activation process by KOH improves the adsorption capacity of the carbon through increasing the surface area and porous structure.

### Adsorption kinetic models

To understand the dynamics of adsorption process of phenol on AC and to describe the adsorption rate, the adsorption kinetic data were fitted to the pseudo-first order and pseudo-second order models. The Lagergren equation (or pseudo first order equation, Eq. 1) and pseudo second order equation (Eq. 2) are represented as follows, respectively:

1 $$\log ({{\rm{q}}}_{{\rm{e}}}-{{\rm{q}}}_{{\rm{t}}})={{\rm{logq}}}_{{\rm{e}}}-\frac{{{\rm{K}}}_{1}}{2.303}{\rm{t}}$$

2 $$\frac{{\rm{t}}}{{{\rm{q}}}_{{\rm{t}}}}=\frac{1}{{\rm{h}}}+\frac{1}{{{\rm{q}}}_{{\rm{e}}}}{\rm{t}}$$

where q_t_ and q_e_ are the adsorbed amount (mg·g^−1^) of phenol at any time t and at equilibrium, respectively. K_1_ is the rate constant for pseudo-first order model (min^−1^) and h (mg·g^−1^ min^−1^) is the initial adsorption rate equal to ${{\rm{K}}}_{2}{{\rm{q}}}_{{\rm{e}}}^{2}$, K_2_ is the rate constant for the pseudo second order model (g mg^−1^ min^−1^). The values of K_1_ and R^2^ at different initial concentrations of phenol were obtained by plotting a graph between log (q_e_ − q_t_) vs t (Table 2). Besides, q_e_ and k_2_ were determined from the slope (1/q_e_) and intercept (1/h) of the straight line of $\frac{{\rm{t}}}{{{\rm{q}}}_{{\rm{t}}}}$ versus t, passing through origin for all three different initial concentrations of phenol (Table 2).

**Table 2:** Kinetic parameters for the removal of phenol by the prepared activated carbon by KOH in temperature of 800 °C and impregnation ratio (KOH to carbon) of 2:1 (CK2T800).

Activated carbon	C_0_ (mg L^−1^)	q_e_ (mg g^−1^) (experimental)	First-order kinetic model			Second-order kinetic model		
			K_1_	q_e_ (mg g^−1^) (calculated)	R^2^	K_2_	q_e_ (mg g^−1^)	R^2^
CK2T800	50	60.2	0.133	59.6	0.960	0.002	74.62	0.993
	100	115.4	0.0907	83.4	0.985	0.0015	136.98	0.998
	200	226.1	0.1087	197.7	0.976	0.0009	263.15	0.997

The experimental data are well described by both kinetic models. However, the correlation of coefficient (R^2^ = 0.99) for three different initial concentrations of phenol is higher for the second-order kinetic model. The values of K_1_ and K_2_ decreased with increasing the initial phenol concentrations (50–200 mg L^−1^) suggesting that the chemisorption was not the only rate limiting factor, and that both physisorption and chemisorption controled the adsorption process^29,30^.

### Adsorption isotherm models

The empirical Freundlich isotherm model supposes that the adsorbent involves no uniform sorption and the sorption of adsorbate occurs under a heterogeneous adsorption system. In contrast, the Langmuir isotherm model describes a monolayer adsorbent containing a finite number of adsorption sites with a homogeneous surface. Temkin isotherm is more useful in describing the adsorption energy distribution on heterogeneous surface, while Dubinin-Radushkevich isotherm is an empirical adsorption model indicating that the adsorption process occurred onto both homogeneous and heterogeneous surfaces^29^. The logarithmic form of the equation of the Freundlich model is expressed as follows:

3 $$\log \,{{\rm{q}}}_{{\rm{e}}}=\,\log \,{{\rm{k}}}_{{\rm{f}}}+1/{\rm{n}}\,\log \,{{\rm{C}}}_{{\rm{e}}}$$

where C_e_(mg L^−1^) is the equilibrium concentration of phenol and q_e_ (mg g^−1^) *is* the amount of phenol adsorbed per gram of AC at equilibrium. K_f_ and n are the *Freundlich model* constants being indicative of adsorption capacity and adsorption intensity, respectively. These values can be determined from the slope and intercept of the *produced straight line* of the linear plot of *log* C_e_ against *log (q*_*e*_*)*, respectively*. The magnitudes of* K_f_ and n present desirable sorption and easy separation of adsorbate from the aqueous solution.

Also, the linearized form of the Langmuir equation is described as follows:

4 $${{\rm{C}}}_{{\rm{e}}}/{{\rm{q}}}_{{\rm{e}}}=1/{{\rm{q}}}_{{\rm{m}}}{{\rm{C}}}_{{\rm{e}}}+1/{{\rm{K}}}_{{\rm{L}}}{{\rm{q}}}_{{\rm{m}}}$$

where q_m_(mg g^−1^) and K_L_(L mg^−1^) are the Langmuir isotherm constants related to the maximum adsorption capacity of the sorbent (mg g^−1^) and the free energy of adsorption (L mg^−1^), respectively. These two values were calculated from the slope (1/q_m_) and intercept (1/K_L_ q_m_) of the line obtained by plotting C_e_/q_e_ against C_e_.

Temkin isotherm has the linear forms as following equation:

5 $${{\rm{q}}}_{{\rm{e}}}={\rm{B}}\,{{\rm{lnK}}}_{{\rm{t}}}+{\rm{B}}\,\mathrm{ln}\,{{\rm{C}}}_{{\rm{e}}}$$

In above equation ${\rm{B}}={RT}/{b}_{t}$, R is the gas constant (8.314 J mol^−1^ K^−1^), T (°K) is the absolute temperature, b is the Temkin energy constant related to the heat of adsorption (J mol^−1^), K_t_ is the Temkin isotherm constant (L g^−1^). The values of b, B and K_t_ are determined from the slope and intercept of linear plot of *q*_e_ versus ln C_e_.

Dubinin-Radushkevich model were calculated from the following equation:

6 $$\mathrm{ln}\,{{\rm{q}}}_{{\rm{e}}}=\,\mathrm{ln}\,{{\rm{q}}}_{{\rm{m}}}-{{\rm{K}}}_{{\rm{DR}}}\,{{\rm{\varepsilon }}}^{2}$$

7 $${\rm{\varepsilon }}={\rm{RT}}\,\mathrm{ln}(1+\frac{1}{{{\rm{C}}}_{{\rm{e}}}})$$

where K_DR_ is a constant related to the mean free energy of adsorption (mol^2^ K^−1^ J^−2^). By plotting ln q_e_ against *ε*^2^, the value of Dubinin-Radushkevich constant can be determined.

All the model parameters were calculated using Origin 8.0 software. The standard errors (SE) for each parameter and the correlation coefficient (R^2^) were used to measure the goodness-of-fit and determined the best-fitting isotherm to the experimental data, respectively (Table 3). Based on the high values of correlation coefficient (greater than 0.99), both Langmuir and Freundlich models described better phenol adsorption onto AC than Temkin and Dubinin-Radushkevich isotherms models. In this regard, Issabayeva et al^31^. described that many studies on the phenol adsorption reported good fitness of the phenol adsorption data into both isotherm models, indicating a mixed adsorption behavior. They also stated that the majority of phenol adsorption studies were in good agreement with better description of adsorption process of phenol by Freundlich isotherm. In this study, although the experimental equilibrium data showed a satisfactory fit to both models, the values of SE for each parameter obtained from Langmuir isotherm model were found to be higher than that of Freundlich isotherm model, as shown in Table 3. This suggests that the phenol adsorption process on the produced AC can be relatively heterogeneous^30^.

**Table 3:** The Freundlich, Langmuir, Temkin, Dubinin-Radushkevich model constants for adsorption of phenol onto the prepared activated carbon.

Models	Parameters				
Freundlich	*K*_*f*_ mg g^−1^) (L mg^−1^)^1/n^	SE	n	SE	*R*^2^
	20.26	0.79	1.15	0.04	0.997
Langmuir	*q*_*m*_ (mg g^−1^)	SE	*K*_*L*_ (L mg^−1^)	SE	*R*^2^
	434.78	35.97	0.047	4.4×10^−3^	0.992
Temkin	b_t_(J mol^−1^)	SE	*K*_*t*_(L g^−1^)	SE	*R*^2^
	50.19	5.45	1.14	0.16	0.967
Dubinin-Radushkevich	*q*_*m*_	SE	K_DR_ (mol^2^ K^−1^ J^−2^)	SE	*R*^2^
	97.42	12.7	7×10^−7^	1.3×10^−7^	0.908

It is also worth mentioning that the the selected AC showed a very high phenol adsorption capacity of 434 mg g^−1^. This value of q_m_ demonstrates that the synthesized AC can be used as an effective adsorbent for the removal of phenol from aqueous solution and also probably of other water pollutants. The well-developed porous structure, microporosity and the surface chemistry of activated carbons are the most important factors affecting the adsorption of phenol onto the adsorbents^32,33^. Table 4 summarizes phenol maximum adsorption capacities and BET surface areas for different ACs reported in the literature. It reveals the high potential of the produced AC in the current study for phenol removal from aqueous solutions in comparison to the other sorbents.

**Table 4:** Comparison of BET surface area and maximum phenol adsorption capacities of various activated carbons (ACs).

Adsorbent	BET (m^2^·g^−1^)	Q_m_ (mg·g^−1^)	Reference
Oily sludge based AC	2263	434	This study
Cattle bone based AC	2687	431	^6^
Rice husk based AC	2138	201	^7^
Soybean straw based AC	2271	278	^8^

### Adsorption mechanism

The affinities of the adsorbent towards the components of interest can be determined from the content of surface functional groups and pH_pzc_. The pH_pzc_ indicates zero net surface charge of the adsorbents that implies their electronic surface charges as well as surface oxygen complexes. At pH values less than the pH_pzc_ (pH < pH_pzc_), water donate more H^+^ than OH^−^ groups, therefore the adsorbent surface has a net positive charge and attracts anions.

The pH_pzc_ of the prepared AC was 7.9 that indicated its slightly basic character. The ACs with basic surface characteristics, such as the adsorbents of this study, are considered as the most appropriate and effective adsorbents for the removal of phenol due to the weak acid nature of this molecule. The adsorption of organic compounds onto the ACs mainly contribute to three types of surface–phenol interactions namely, (i) π-π dispersion interaction, (ii) the electron-donor–acceptor complex formation and (iii) the hydrogen-bonding formation^34^ that may occur simultaneously. Surface oxygen complexes sites are of Bronsted type. The appearance of acidic functional groups such as carboxyl and phenolic groups, due to oxidation under aerobic conditions, causes the π electron to be removed from carbon matrix and decreases the amount of adsorbed phenol from the solutions. But the increase in the surface basicity of the activated carbon due to the fixation of surface oxygen groups of basic character during chemical activation favors the formation of electron donor acceptor complex. The existence of surface oxygen groups located at π-electron-rich regions provide the formation of mentioned complexes with aromatic rings of phenol. These Lewis basic centers predominate on the surface of adsorbents with low oxygen content. During the adsorption of phenol on activated carbon, these regions act as donor and the aromatic rings of phenol as acceptor. Phenol adsorption onto the carbons is controlled by dispersive force between π electrons^35^.

Beside the surface chemistry of activated carbon, pore structure also affects the adsorption process. The porosity of carbonaceous material has been considered as an important factor in adsorption processes of organic compounds from aqueous solutions. It has been reported that the adsorption capacity of small molecules such as phenol to the inner surface of carbon correlates with the content of micropores and BET surface area, while for mesoporous ACs, substituent group in the phenol and nature of the carbon controlled the phenol adsorption as well^35^.

### Thermodynamics

The influence of adsorption temperature (25 to 40 °C) on the adsorption of phenol was investigated. It can be observed that the adsorption of phenol decreases with increasing temperature. Thus, the maximum adsorption was achieved at the lowest adsorption temperature (25 °C). This probably indicates a poor chemical reaction interplay between adsorbate and surface functionalities of AC^36^.

Thermodynamic parameters such as free energy change (∆G°), enthalpy (∆H°), and entropy (∆S°) were estimated according to the following equations:^37^

8 $$\varDelta G=-RTln{K}_{d}$$

9 $${K}_{d}=\frac{{q}_{e}}{{C}_{e}}$$

10 $$\varDelta G=\varDelta H^\circ -T\varDelta S^\circ $$

where R is the gas constant (8.314 J·mol^−1^ K^−1^), T is the temperature (K), K_d_ is the distribution coefficient. q_e_ and C_e_ are the equilibrium concentrations of phenol on the adsorbent (mg L^−1^) and in the solution (mg L^−1^), respectively.

The values of ∆H° and ∆S° were obtained from the slope and the intercept of plot of ln K_o_ versus 1/T for initial phenol concentrations of 50, 100 and 200 mg·L^−1^. The thermodynamic parameters are given in Table 5. The negative ∆G° values were observed in all cases, indicating the spontaneous nature of phenol adsorption onto AC. In addition, the decrease in the magnitude of ∆G° at the higher temperatures showed the diminishing of the spontaneous of the process so the adsorption was not favorable at higher temperatures. Moreover, it was observed that all the values of ∆H° were negative demonstrating the exothermic nature of the adsorption interaction which was also supported by the experimental observations. The decreased randomness at the solid-solution interface during the sorption of phenol on AC is well explained by the negative value of the entropy^38^.

**Table 5:** Thermodynamic parameters for the adsorption of phenol onto the produced activated carbon at pH 6.

Temperature (°k)	C_0_ (mg L^−1^)	q_e_ (mg L^−1^)	ln K_0_	∆G (kJ mol^−1^)	∆H (kJ mol^−1^)	∆S (J kmol^−1^)
298	50	59.57	3.23	−7.91	−79.83	−241.33
303	50	56.77	2.51	−6.707		
308	50	54.45	2.13	−5.5		
313	50	50.17	1.62	−4.29		
298	100	112.58	2.42	−5.85	−55.97	−168.16
303	100	104.35	1.84	−5.01		
308	100	100.55	1.63	−4.17		
313	100	92.65	1.27	−3.33		
298	200	216.52	2.17	−5.29	−54.55	−165.29
303	200	199.8	1.66	−4.46		
308	200	185.1	1.4	−3.64		
313	200	170.2	1.07	−2.81		

### Removal of phenol from real industrial wastewater

Phenol adsorption capacity from industrial wastewater was obtained 90.11 mg·g^−1^. The result indicated that, as expected, the phenol removal efficiency of this AC in synthetic wastewater was greater than in real effluents. This is probably due to the pH of real effluents as well as the presence of other chemical species and impurities in textile effluent that could interfere and/or compete in the process of adsorption.

### Regeneration studies

**Figure 5:**
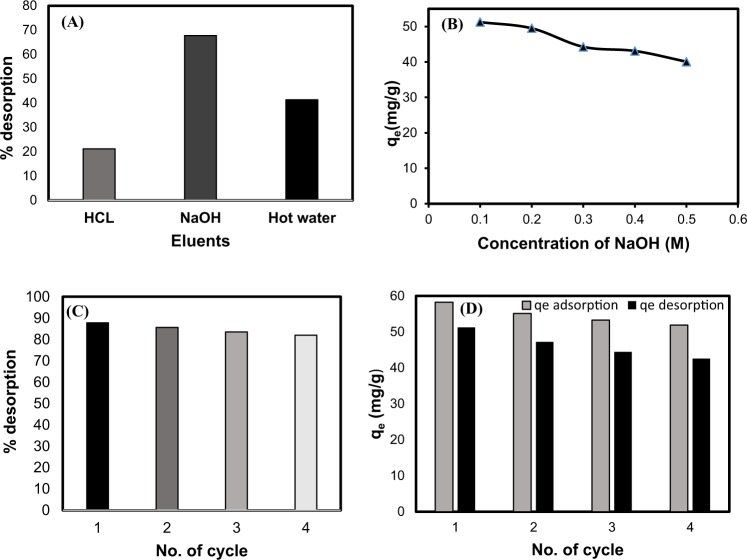
Comparison of different eluents for regeneration (**A**), effect of different concentrations of NaOH on desorption of phenol from the activated carbon (**B**), and sequential adsorption–desorption cycles of the activated carbon for phenol removal using 0.1 M NaOH (**C** and **D**).

The reversibility of the adsorption process and the feasibility of adsorption and desorption behavior of the AC for the phenol removal were examined using hot water, HCl and NaOH solutions. The better regeneration performance of the NaOH solution compared to the HCl solution (Fig. 5(A)) can be explained by the fact that phenol react with NaOH, resulting in the formation of soluble salt C_6_H_5_O^−^Na^+^ which facilitates the desorption of phenol from the adsorbent^39^.

The higher desorption amount obtained when using NaOH solution compared to hot water could be due to 3 simultaneous mechanisms: (1) hydrolysis of some chemical bonds between phenol hydroxyl groups (−OH) and surface oxygen groups of the adsorbent, (2) higher solubility of phenol (reactive species under alkaline conditions) than that of neutral molecules, and (3) favorable desorption of phenol in NaOH solution due to deprotonation of acidic groups of the adsorbent surface and adsorbed phenol, as well as increasing electrostatic repulsion between these molecules^40,41^. As shown in Fig. 5(B), 0.1 M NaOH was found to be the best desorbing agent for regenerating the AC. Ozkaya^42^ also reported that more than 60% phenol was desorbed from the AC using 0.15 M NaOH solution.

In order to investigate the adsorbent regeneration ability, consecutive adsorption-desorption cycles were repeated four times using recycled AC as the regenerated sorbent and 0.1 M NaOH as the effective eluting agent (Fig. 5(C,D)). As can be seen, the synthesized AC maintained most of its adsorption capacity through successive adsorption-desorption cycles.

## Conclusion

Highly microporous activated carbons were prepared from oily sludge using two-step method under different synthesis conditions. The optimal activated carbon that obtained at activation temperature of 800 °C and impregnation ratio of 2:1 (KOH: precursor) had unique texture structure such as, high BET surface area, well-developed pore structure and high volume of micropores and total pore volume. Phenol adsorption experiments showed a maximum adsorption capacity of 434 mg g^−1^. The Freundlich isotherm showed the best fit to the equilibrium experimental data. The kinetic data were fitted well to pseudo-second-order kinetic model. Since removal of phenol as a weak acid was pH dependent and better in basic medium, the oily sludge derived activated carbon with basic surface characteristic and porous structure showed effective adsorbent for phenol treatment from aqueous solution. The negative values of both free energy change and enthalpy change implied the spontaneous and exothermic nature of the adsorption process. The 0.1 M NaOH solution was the most effective desorbing agent for adsorbed phenol from loaded AC. Regenerating of AC demonstrated that the produced sorbent could be reused for phenol removal repeatedly from aqueous solution without significant loss of adsorption capacity. The results of this work demonstrated the potential use of oily sludge as a low cost precursor for production of AC with a high surface area, well-developed porosity and considerable adsorption properties. Further studies are necessary to assess the environmental and economic aspects for using this process in the pilot scale.

## Experimental

All chemicals used were supplied by Merck Company (Germany). The Oil Refinery Sludge (ORS) was collected from crude oil storage tanks of Isfahan Oil Refinery, Iran.

### Characterization of raw material

The ash content of oily sludge was measured using the method of ASTM D 482–487^43^. CHNS elemental analyzer (Elementar Vario EL III) was used for measuring the carbon (C), hydrogen (H), nitrogen (N), and sulfur (S) contents of the oily sludge.

### Synthesis of activated carbons

The oily sludge (100 g) was initially pyrolyzed in the absence of activating agent in a vertical furnace at a temperature of 500 °C, based on the TGA analysis, with a heating rate of 10 °C min^−1^ for 1 hours, under nitrogen atmosphere with flow rate of 250 cm^3^ STP min^−1^. Afterwards it was cooled down to the ambient temperature (thermal treatment). In the second stage (thermochemical treatment), ACs were prepared at different activation temperatures and impregnation ratios (mass ratio of activating agent to produced char). The specified weight of ORS char (approximately 5 g) was physically mixed with corresponding weight of KOH at different impregnation ratios of 1:1, 2:1 and 3:1 in a ceramic crucible for 24 hours. The mixture was heated at different activation temperatures of 600, 700, 800 and 900 °C under the same N_2_ flow and heating rate for the first step. The obtained products were successively washed. They were stirred several times in 1 liter of boiling distilled water and subsequently 1 liter of cold distilled water followed by 1 L of HCl (0.1 M) until the pH of outlet liquid was nearly 7. Finally, the samples were dried overnight at 60 °C in oven. The synthesized ACs were denoted as CK followed by the impregnation ratio and the activation temperature (e.g. CK2T700).

### Characterization of prepared activated carbons

The yield of the activated carbon was estimated according to the following equation:

11 $${\rm{Yield}}\,({\rm{wt}}. \% )=({{\rm{W}}}_{{\rm{f}}}/{{\rm{W}}}_{{\rm{o}}})\times 100$$

where W_f_ and W_o_ are the dry weight of the produced activated carbon and the initial dry weight of carbonized oily sludge, respectively. The iodine number was measured by the standard test for determination of iodine adsorption number (ASTM D 4607-94^44^). To investigate the specific surface area, pore volume, and pore diameter of the obtained activated carbons, N_2_ adsorption-desorption at −196 °C was performed in a Micromeritics TriStar II 3020 apparatus at sub atmospheric pressures. The micropore volume was calculated by using t-Plot micropore volume. The samples were previously degassed under vacuum at 150 °C for at least 8 h. Brunauer-Emmett-Teller (BET) method was applied to quantify the total surface areas (S_BET_). The t- plot method was used to determine the micropore volume, V_mic_, micropore surface area (S_mic_), external or non-microporous area (S_ext_), and the mean micropore diameter (D_mic_). The pore structure and surface morphology of the obtained AC with the highest surface area was studied by scanning electron microscopy (SEM, Tescan, VEGA III) after gold coating of the sample. Fourier transform infrared (FTIR) spectroscopy (Tensor 27, Brucker) was used to identify the surface functional groups of the AC with the most developed porosity in the wave number range of 4000 to 400 cm^−1^. The sample was mixed with KBr at a 1:20 ratio and pressed to prepare the pellet.

### Determination of pHpzc

To quantify the point of zero charge (PZC), certain amounts of AC were introduced into 0.1 mol L^−1^ NaCl solutions. The pH of the solutions was adjusted from 4–10 using dilute solutions of HCl (0.1 M) or NaOH (0.1 M). The bottles containing the suspensions were sealed and continuously shaken for 48 h and the final pH were measured.

### Phenol adsorption tests

Efficiency of the synthesized ORS char and ACs were tested as adsorbents for the phenol sorption from aqueous solutions in the batch experiments. All experiments were duplicated with control experiment in the same conditions without adding the adsorbent. Initial experiments were performed using 1 g of AC per liter added to the phenol solution (100 mg L^−1^) with natural pH of prepared solutions in the polyethylene flasks. The suspension was stirred for 4 h at 25 °C. After filtration through 0.45 μm syringe filters, the concentration of phenol in the aqueous phase was analyzed using ultraviolet visible (UV-VIS) spectrophotometer (Varian, Model Cary 1E) at 270 nm wavelength. The AC with the highest phenol adsorption capacity and BET surface area was used for further batch adsorption experiments to study the influence of contact time (t), initial phenol concentration (C_0_), initial pH (pH_0_) and adsorbent dose (m) on phenol removal from aqueous solution. All experiments were run with a known mass of adsorbent in 50 mL phenol solutions and were shaken in a shaking water bath (25 °C) with constant speed of 200 rpm. At the end of the adsorption time, the content of each flask was filtered and phenol concentrations in the solutions were determined. The adsorption capacity (q_e_) was determined by the following equation:

12 $${{\rm{q}}}_{{\rm{e}}}=\frac{({C}_{0}-{C}_{e})V}{m}$$

where C_0_ and C_e_ are the initial and final concentration of phenol (mg L^−1^) in solution, respectively, q_e_ is the adsorption capacity of phenol by the adsorbent (mg of phenol per g of AC), m is the mass of activated carbon (g) and V is the volume of phenol solution (L).

The adsorption kinetics were done following a similar procedure at temperature of 25 °C and natural pH in three initial phenol concentrations of C_0_ = 50, 100 and 200 mg L^−1^. The samples were detached at predefined time spacing and phenol uptake at time t was determined using Eq. 13.

13 $${{\rm{q}}}_{{\rm{t}}}=\frac{({C}_{0}-{C}_{t})V}{m}$$

where C_t_ (mg L^−1^) is liquid concentration of phenol at any time.

In this study, the adsorption equilibrium data were analyzed using four types of sorption isotherm models including Langmuir, Freundlich, Temkin and Dubinin-Radushkevich. To investigate the adsorption isotherms, the experiments were conducted by adding a known amount of adsorbent (0.8 g L^−1^) to the phenol solutions with different initial concentrations of C_0_ = 20–100 mg L^−1^ at 25 °C and equilibrium time.

Furthermore, to elucidate the effect of temperature on the adsorption, 0.8 g L^−1^ adsorbent dose was added to each sample contains 50 mL of phenol solution with different initial concentrations of 50, 100 and 200 mg L^−1^. The suspensions were agitated at 200 rpm for 1 h in a water bath shaker at different temperatures in the range of 25 to 40 °C.

### Adsorbent regeneration

For the purpose of regenerating the adsorbent, various solvents including HCl, NaOH and hot water were used for the elution of phenol from the AC. The AC loaded with phenol, obtained after the adsorption process under optimized conditions, was added into 50 mL of eluent solutions. The samples were shaken (200 rpm at 25 ± 2 °C) for 3 hours. Then the effluent was analyzed to determine the concentration of desorbed phenol. The adsorption/desorption procedure of AC with the best desorbing agent were repeated in the four cycles on the same matrix to study the reusability of the AC. The percentage of desorbed phenol was calculated by the following equations:

14 $${\rm{Desorption}}\,( \% )=[\frac{{q}_{des}}{{q}_{ads}}]100$$

15 $${q}_{des}={C}_{des}\frac{V}{W}$$

where, q_des_ is the content of desorbed phenol (mg g^−1^) and C_des_ (mg L^−1^) is the concentration of phenol in the solution with volume V (L) and W is AC weight (g).

## Data Availability

All data generated or analyzed during this study are included in this manuscript.
